# Long-Term Impact of Disasters on the Public Health System: A Multi-Case Analysis

**DOI:** 10.3390/ijerph17176251

**Published:** 2020-08-27

**Authors:** Nina Lorenzoni, Verena Stühlinger, Harald Stummer, Margit Raich

**Affiliations:** 1 Department for Public Health, Health Services Research and Health Technology Assessment, UMIT TIROL—Private University for Health Sciences, Medical Informatics and Technology, 6060 Hall in Tirol, Austria; verena.stuehlinger@umit.at (V.S.); harald.stummer@umit.at (H.S.); margit.raich@umit.at (M.R.); 2 Department of Business, University Seeburg Castle, 5201 Seekirchen am Wallersee, Austria

**Keywords:** long-term impact, disaster, public health, case study, disaster management, multi-case analysis, Europe

## Abstract

As past events have shown, disasters can have a tremendous impact on the affected population’s health. However, research regarding the long-term impact on a systems level perspective is still scarce. In this multi-case study, we analyzed and compared the long-term impacts on the public health system of five disasters which took place in Europe: avalanche (Austria), terror attack (Spain), airplane crash (Luxembourg), cable-car tunnel fire (Austria), and a flood in Central Europe. We used a mixed-methods approach consisting of a document analysis and interviews with key stakeholders, to examine the various long-term impacts each of the disasters had on health-system performance, as well as on security and health protection. The results show manifold changes undertaken in the fields of psychosocial support, infrastructure, and contingency and preparedness planning. The holistic approach of this study shows the importance of analyzing long-term impacts from the perspective of the type (e.g., disasters associated with natural hazards) and characteristic (e.g., duration and extent) of a disaster, as well as the regional context where a disaster took place. However, the identified recurring themes demonstrate the opportunity of learning from case studies in order to customize the lessons and apply them to the own-disaster-management setting.

## 1. Introduction

The impact of disasters on the health of the people affected and on the public health system can be far-reaching and long lasting. However, studies examining these long-term influences months or even years (mid- to long-term periods) after the event are rare, although their importance is repeatedly underlined [1,2,3]. Nomura et al. (2016) emphasize the need for a better understanding of long-term health impacts of disasters, in order to be able to set measures and guide actions (before, during, and after the event) to reduce health risks [4]. Moreover, the Sendai Framework for Disaster Risk Reduction points out, in Priority 4 (“Enhancing disaster preparedness for effective response and to ‘Build Back Better’ in recovery, rehabilitation and reconstruction”), that the importance of post-disaster reviews as they offer a valuable source for learning lessons for the public health system and consequently raise disaster preparedness [5]. Such a systematic review of challenges, dysfunctions, and, consequently, changes, as well as impacts, would be essential to improve preparedness for future events [6]. A better understanding of the impacts of disasters on the public health system and which determinants influence these impacts would be an important contribution to reduce disaster risk. The Sendai Framework advocates for the collection and analysis of the impacts of disasters and consequently the dissemination of the resulting lessons learned across all relevant stakeholders. Researchers are asked for their contributions, sharing their evaluations with practitioners, government officials, and policy makers, to support decision-making and the implementation of good practices on local, regional, and national level [5]. Especially studies on mid- to long-term periods, i.e., months to years after the event, would be needed. A long-term study is difficult, since data are hardly available in a consistent form, and the quality of these data also varies greatly depending on jurisdiction and time frame [7]. Furthermore, the literature review provided no recommendations for the definition of “long-term”. The studies differed in the use of time spans, but no discussion was found as to why the authors used specific time spans.

Investigations on the long-term impact of disasters on the health of people affected cover a wide field of research topics (e.g., disaster impacts such as economic losses of the affected population or effects on mental and/or physical health; or focus on specific target groups such as children or the elderly [8,9,10,11,12,13]). Research on economic and human loss or damage tends to focus on short-term effects, while longer-term effects are difficult to track, and public attention often shifts on the next disaster event [4,14]. Murthy et al. [15] analyzed the progress made in public health preparedness within the US after 9/11, until 2016, by using self-reports from “Centers for Disease Control and Prevention” and reports of congress funding. Fitter et al. [16] examined the recovery of the public health system of Haiti following the 2010 earthquake and cholera outbreak. By using a framework consisting of 10 essential public health services, their research demonstrated progress and improvement made regarding the public health system seven years after the disaster. Our literature review revealed that the research on long-term impact mainly focuses on individual or population health, but less on the systems perspective. 

The purpose of this study was to investigate the various long-term impacts on the public health system of five different disasters occurring in Europe, from 1999 to 2013. The chosen holistic research approach allowed the representation of the complexity, specific circumstances, and undertaken measures for each disaster in detail. This process allowed us to identify undertaken adaptions and changes that result in longer-term impacts.

## 2. Materials and Methods 

### 2.1. Design

We decided to use a case study approach, as it allowed for us to capture both the diversity in disaster management and the complexity of specific circumstances. An extensive collection of rich data makes an embedded multiple-case analysis and presentation possible. The selection of multiple cases resulted from replication logic [17]. We built upon contrasting cases [18], since a variety of contexts, circumstantial factors, and their impact on each case could offer a more complete picture of the longer-term impact of disasters on public health. Since the study was conducted within the EU project PsyCris (PSYchosocial support in CRISis Management), the selected case studies all took place in the European Union. The project consortium decided to use disasters, which are relevant for many EU countries. In addition to the high probability, further criteria for the decision of which cases to use were the scale of the disaster, complexity and number of institutions involved, long-term consequences, and data availability. Moreover, the analysis should include disasters caused by natural hazards, as well as human-caused disasters (e.g., technological or mass-violence).

We decided to use a mixed-method approach consisting of document analysis and expert interviews. As we could not identify a standardized assessment tool to investigate the long-term impact of disasters to the public health system in the literature, we decided to focus on impact models [19,20,21,22] that serve as a foundation for the development of the interview guide and the category grid used for the document analysis. 

The main categories in the grid were as follows:

- Information about the chosen disaster (what happened, number of people affected, which organizations were involved in disaster management and response, and reaction of the healthcare system);
- General coping strategies and direct effects on health;
- Direct costs and follow-up costs;
- Long-term effects on the public healthcare system;
- Long-term effects on culture and the community.

For our analysis, we reviewed key documents relevant to the disaster-management process and the long-term impact of chosen disasters. The reviewed documents included governmental and organizational reports, legislative documents, journal articles, books, letters, TV documentaries, brochures, and newspaper articles. 

As a reconstruction of the complex disaster management process is not possible with existing public documents only, we also conducted expert interviews. 

The interview partners all played key roles in the management of the respective disaster. When selecting the interview partners, care was taken to obtain a picture as broad and holistic as possible of the most diverse organizations and units. The professional fields of the interview partners included fire department, emergency psychologists, politicians, armed forces, police, physicians, red cross, forensic, and priests. The average interview duration was 60 min. Table 1 shows the number of interviewees per case study. 

The main themes of the interviews were as follows:

- Job description and responsibilities of the interviewed person;
- Description of the event and the disaster-management process;
- Organization and cooperation of rescue organizations and teams;
- Psychosocial support and challenges for the disaster managers;
- Community resilience;
- Security/protection of population.

For the interview analysis, we used GABEK^®^ (Holistic Processing of Complexity) (Josef Zelger, Innsbruck, Austria), a qualitative method of knowledge organization developed by Josef Zelger, supporting analysis of unstructured texts on the basis of the theory of “linguistic gestalten”. The method helps to handle individual thoughts and attitudes and present them in a structured and systematic way. GABEK allows for the transparent organization of knowledge and captures the holistic representation of complex social situations, like disasters, from the perspective of those affected [23]. The analysis was conducted with the corresponding software application GABEK–WinRelan, which combines qualitative content analysis (e.g., coding of keywords, evaluations, or causal relationships) and quantitative measures (e.g., frequencies of keywords and relations) and therefore offers a profound understanding of data, their interlinkages, and their weight [24].

### 2.2. The Case Studies 

#### 2.2.1. Avalanche, Austria, 1999

On 23 February 1999, shortly after 4:00 p.m., an avalanche buried around 100 people in the village of Galtuer (Austria). Ongoing snowfall in the previous days triggered high avalanche risk in the entire federal state of Tyrol. The Paznaun valley, in which Galtuer is located, was already cut off from the outside world before the avalanche, due to the snow conditions. The avalanche hit areas in the village center, which had previously been considered safe from avalanches. Due to the bad weather conditions, no rescue teams were able to reach Galtuer for assistance. The people affected were left to their own devices to rescue the buried victims. Only on the 24 February did the first helicopters could take off and bring the injured from the village to the surrounding hospitals. The Austrian government asked NATO (North Atlantic Treaty Organisation) and neighboring countries for support with additional helicopters. On 25 February, aerial evacuation of the whole valley began with 37 helicopters from Austria, Germany, Switzerland, France, and the US. Thirty-one people died in the avalanche, and 35 were injured (11 of them severely). 

#### 2.2.2. Cable-Car Accident, Austria, 2000

In Kaprun (Salzburg), on 11 November 2000, in the tunnel of the cable-car, a fire occurred in the ascending train. The glacier around the Kitzsteinhorn is a popular all-the-year skiing region. On 11 November, the train started at 8:57 in the morning, with 161 passengers aboard. At 9:02 a.m., the first smoke formed. At 9:05 a.m., the train stopped after 600 m in the tunnel; the total distance from the entrance to the exit was 3.8 km. Passengers broke the windows. Smoke rose from the train at the back. The operator saw the fire and gave alarm. Twelve passengers walked with their ski boots downstairs and survived. The other 149 passengers and the operator were in the tunnel. At 9:35 a.m., because of the danger of explosion, the outfall of electricity, and the formation of toxic smoke, the fire brigade had to stop the rescue mission. The mountain station had to be evacuated. At 10:16 a.m., a rescue team started to enter the tunnel. Three people died at the mountain station because of exhaust-gas poisoning. One person survived. At 12:00 p.m., a second team of the fire brigade entered the tunnel. In total, 155 passengers died on 11th of November.

#### 2.2.3. Airplane Crash, Luxembourg, 2002

On Wednesday, 6 November 2002, an airplane of the national air company crashed during the approach to Luxembourg airport. At 10:05 a.m., the airplane reported difficulties during the landing procedure, and 42 s later, the plane disappeared from the radar. The reasons for the airplane crash are based on a mix of technical problems, human errors, and bad weather conditions. At 10:06 a.m., the plane ploughed into a field, and one side of the plane was ripped open. Six passengers were catapulted out. After the crash, the aviation fuel caught fire, and the plane began to burn. Both pilots were trapped in the wreckage. After fire brigades had extinguished the fire, the rescue of the trapped passengers began. The only survivors had been sitting in the front of the aircraft, which had been torn off during the crash, leaving the nose cone embedded in the ground. Twenty people died and two survived severely injured.

#### 2.2.4. Terror Attack, Spain, 2004

On 11 March 2004, ten explosions occurred aboard four commuter trains in the city of Madrid. It was later reported that thirteen improvised explosive devices had been hidden on the trains. The explosions happened during rush hour, on a normal workday, between 7:37 a.m. and 7:39 a.m. At 7:37 a.m., three bombs exploded on train number 21,431, on track two, inside Atocha station, the main capital railway station. At 7:38 a.m., two bombs exploded in train number 21,435, at El Pozo del Tío Raimundo Station. One bomb exploded on train number 21,713, at Santa Eugenia Station, at 7:38 a.m. Then, at 7:39 a.m., four bombs exploded on train number 17,305, on Calle Téllez, approximately 500 m from Atocha station. The bombing was the deadliest terror attack in the history of Spain: 191 people died, and more than 2000 were injured. 

#### 2.2.5. Flood, Austria/Germany, 2013

Due to exceptionally heavy rainfalls in early June 2013, extreme flooding of the major river systems occurred throughout Central Europe (particularly Switzerland, Germany, Austria, Czech Republic, Slovakia Hungary, and Serbia). Thousands of people needed to be evacuated from their houses. Twice as much rainfall as average during the month in Austria resulted in the soil becoming saturated. Although forecasts predicted a high rise of water levels, the prognoses have been too low. The water level exceeded the levels seen during the disastrous “once in a century” Central European floods of 2002 in many areas. In Austria, six people died in the flood, and thousands of people had to be evacuated. The infrastructural damages were extensive; railway lines, roads, bridges, and houses were damaged severely. 

## 3. Results

**Figure 1:**
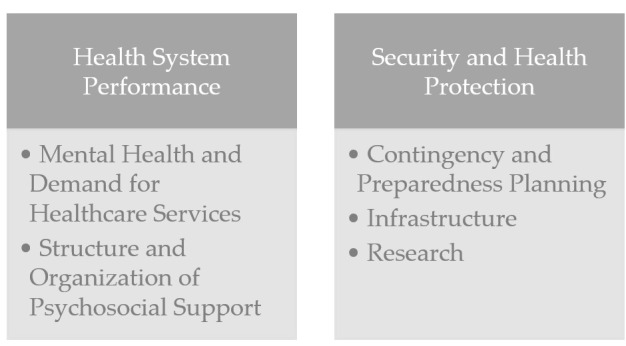
Overview of the aggregated long-term impacts of the analyzed disasters on the public health system (source: the authors).

The following section describes the long-term effects of the disasters on the public health system. Figure 1 gives an overview of the framework of analysis. We decided to categorize the identified impact factors into the categories health system performance, and security and health protection. The increased demand during and after a disaster has a direct effect on the health system performance, i.e., the delivering of services, the creation of resources, the stewardship, and, finally, the related financing [25]. Security and health protection refers to post-disaster efforts aiming at optimizing leadership and governance, contingency and preparedness planning, infrastructure, and training, or may lead to an increase in security research funding activities, as well as information and communication activities. The information stated in this section was extracted from the stated sources, as well as from the expert interviews. 

### 3.1. Long-Term Impact on Health System Performance

#### 3.1.1. Mental Health and Demand for Healthcare Services

For an adequate public health policy, different dimensions have to be taken into account to optimize the offer for healthcare services, including mental health. The identification and evaluation of how many people are affected by a disaster and develop adjustment disorders because of distress seems to be a challenge for health service providers. For the chosen disasters, statistics about the health status of people affected and derived demand for healthcare services are rare.

##### Distinction of Target Groups 

For an analysis of the demand for healthcare services, a distinction of target groups has to be undertaken. Standardized programs for psychosocial and psychological support are not sufficient, as one crisis manager from Luxembourg stated. The demand for psychosocial and psychological support, but also for medical and physical treatments, mainly differs depending on who is affected. The target groups have to be defined carefully, to guarantee adequate support. A study conducted by the Complutense University of Madrid, with 526 victims of the bombings in Madrid, shows that, ten years later, almost 30% of them presented symptoms of anxiety, depression, and PTSD (posttraumatic stress disorder). Ten years later, nearly 200 victims still received psychological treatment [26]. Moreover, a care program has been launched for people with hearing disabilities after the terror attack in Madrid. The explosions and the effects of the blast caused hearing impairments resulting in total deafness in many victims of these attacks. The General Directorate of Support for Victims of Terrorism taught sign language to victims of these attacks and to their families to help them recover communication within their families [27].

##### Psychosocial Support for Specific Target Groups 

An important lesson that has been identified in all cases is the demand for performance-linked psychosocial support. Target-oriented healthcare delivery has to consider the different needs of people affected (e.g., survivors, families, witnesses, and volunteers). Standardization in the provision of services is seen critically by most of the interview partners. One interview partner from Luxembourg explained: “The pilot survived, there was certainly a different need, with the family, than with those where people died; I also think that people had different approaches to dealing with the accident… To accommodate these different “points of view”, that’s extremely difficult. It is often the case that you think you have a solution to a problem, but actually you have to realize in retrospect that you need many solutions for many types of people…and you also have to give people the freedom to deal with the problem in their own way which is enormously difficult”. Because of the fact that nearly all people in the airplane died, the psychosocial support for families played the most important role. The challenge for the disaster management team was the information management to the relatives of the victims, the organization of their arrivals and accommodation, and how to offer psychosocial support. 

Compared to our analyzed disasters associated with natural hazards (avalanche and flood), only a small number of passengers survived in the case of the airplane crash or cable-car accident. The main target group for psychosocial support was the relatives who had to arrive from longer distance or respectively from abroad. Locals and tourists have been identified as important relevant target groups for psychosocial and psychological support after the avalanche. Results have shown that the cohesiveness of the community in Galtuer (=locals) had an essential impact on the demand for psychosocial and psychological support.

The terror attack in Spain, with its destructive power for humans and infrastructure, caused a large number of deaths and people injured. Because of the characteristic of the disaster that occurred in this urban area, more walk-in volunteers entered the scene during the acute phase of the disaster, for help. This has an effect on the demand of psychosocial and psychological support in order to consider a large number of walk-in volunteers.

##### People Affected Who Originate from Abroad

Additionally, in some of the analyzed disasters, no information about psychosocial and psychological support, as well as medical or physical treatment of people who originate from other countries, was given. Especially in the case of the airplane crash, the avalanche and the cable-car accident many victims came from abroad. They were tourists or traveling persons. Survivors and relatives stayed for a certain period time in the country of the event. This leads to the inability to diagnose disorders and reactions, especially of those who were not directly affected by the disaster since some diagnoses cannot be identified immediately after the event, but occur later on (e.g., flashbacks and posttraumatic stress reaction). About 100 of the evacuated tourists in Galtuer were traumatized seriously, and some eventually were confronted with posttraumatic stress disorders later on [28,29].

##### Refusal of Psychosocial or Psychological Support

Individuals or a community may refuse psychosocial or psychological support. An interview partner involved in psychosocial support after the terror attack in Spain explained the following: “I’ve learned not to take it personally but as a normal reaction to emotional trauma. Sometimes we had to work with volunteers who were affected by the comments of relatives, like “if you’re not going to return my son to me I don’t know what you’re doing here… Emotional trauma can cause aggressive reactions and that’s normal”. Some families who lost relatives during the airplane crash in Luxembourg refused psychosocial support. One survivor of the avalanche was buried for several hours. He preferred to talk with his family to cope with the disaster. It was reported that only one woman with adjustment disorders consulted a psychologist after the avalanche disaster in Galtuer [30]. 

A community may also refuse psycho0social and psychological support. In Galtuer, locals dealt intensively with the event and experienced common grief. In the first year, they talked among themselves about the disaster and about their experiences, until they felt some relief from their burden. From the cultural point of view, the locals preferred to talk with family, relatives, or friends, but not with outsiders. Locals also had negative associations with psychologists because of two reasons. First, they experienced an insufficient management of psychosocial support directly after the event. Second, a big distance from and distrust of mental healthcare services were observed. The trust into the community was more helpful for the locals to recreate a meaningful life, as compared to professional organized support. 

##### Risk Perceptions

Results gave interesting insights in the awareness of possible causes of risks. People living in endangered zones may perceive possible causes of risks induced by predictable disasters (e.g., risk of a flood or avalanche) as neutral compared to events that arise abruptly without any advanced warning (e.g., airplane crash or terror attack). The resistance of people living in an endangered zone can be influenced of their motivation and individual risk perception living in such an area. This conscious decision has an impact on the mental and physical health in the context of a disaster. The persons may dispose of a higher acceptance of the forces of nature, given the fact that the inhabitants of Galtuer do not evaluate the avalanches negatively [30]. Moreover, in the investigated flood case, in an area that is prone to flood, people are used to dealing with the flood. One interview partner explained the following: “The people down there, they can handle the water... they have their own strategies, they can handle it. And the humility and the acceptance with which they take the flood there, that’s fascinating for me. I couldn’t imagine that every 10 years I clean out my house, clear out the silt, pump out the water. For them, it’s just the water, it’s as simple as that”. Moreover, most people had family or friends close by with whom they could stay during evacuation, when needed. These two factors are assumed to be a huge reduction of stress for the effected people.

##### Legal Proceedings

A relevant aspect in dealing with disasters is the way of legal response, especially in the case of organizational failures. One important impact on health was identified in the context of legal proceedings after the cable-car accident. Many family members of the victims complained about the lack of empathy during the legal proceedings. The trial was experienced as unfair by many families, leading to a lot of anger and disappointment. The need for clarity and mental processing was not fulfilled [31,32]. During the trial, a self-help initiative was founded which fought for years for resumption of the legal process [33]. Moreover, after the airplane crash, the legal proceedings lasted over years. One interview partner said the following: “[These] court proceedings have simply taken far too long, and that is disastrous for the people who are affected, who simply want decisions and that they can finish their mourning at some point. It has simply taken far too long”.

#### 3.1.2. Structure and Organization of Psychosocial Support

Different longer-term impacts in the structure and organization of psychosocial support have been identified. In one case, the formation of a new organization was induced; in other cases, major or smaller organizational improvements were undertaken. After the avalanche, the disorganization of psychosocial support caused enormous costs for the life assurances, public authorities, and governments. Good management of psychosocial and psychological support, distinct functional attributions, structures, and responsibilities lead to the reduction of conflicts between organizations and institutions involved in the management of a disaster. After the disaster, a discussion was started with regard to financial responsibilities of long-term mental treatments. In the context of psychosocial support, the responsibilities and financing structures for further events were revised. 

##### Formation of New Organizations and Units

A completely new organization for psychosocial support was formed after the Galtuer avalanche. The management of psychosocial support was not organized by one central responsible body. Competition between psychologists and psychotherapists was the source of many conflicts. Additionally, journalists disguised themselves as psychologists and psychotherapists to access the disaster scene. In the years following the disaster, the Austrian Red Cross established the crisis intervention team (KIT—Kriseninterventionsteam). Moreover, it was decided to provide uniforms for the emergency psychologists as they were not associated as professionals by the surviving dependents. An interview partner who was in Galtuer after the avalanche explained the following: “Today it is taken for granted that already the emergency doctor asks if you want a crisis intervention team. But it was different in the past. We didn’t wear a uniform, that was a big problem. And nobody knew the service. The care of uninjured survivors simply didn’t exist”. Although Kaprun, where the cable-car accident happened, is located in Salzburg, in the neighboring province of Tyrol, no structure for psychosocial support existed there at the time of the disaster. However, the tunnel fire was seen as trigger event to also establish a crisis intervention team in Salzburg. In the context of psychosocial support, the extent of the event has led to the formation of a special psychological care unit which takes over the organization and management of psychosocial support. Because of the huge dimension of the terror attack, a systemized supply of psychosocial support for rescue workers in their organizations was established. The awareness for the necessity of psychosocial support for people affected (victims, relatives, and also rescue teams) has grown after all of the analyzed disasters.

### 3.2. Long-Term Impact on Security and Health Protection 

#### 3.2.1. Contingency and Preparedness Planning 

##### Plans and Checklists

An important long-term impact that was identified in all analyzed disasters is the update of existing emergency, civil protection, and national rescue plans and the development or adaption of checklists. Protocols for different contexts have been developed (e.g., intervention protocols for psychological intervention), as well protocols for recruiting people to participate in emergency volunteer management and psychological interventions. The emergency plans and the interfaces between the participating organizations were modified to improve the cooperation and coordination between security teams, armed forces, medical teams (doctors and nurses) in hospitals, social workers, and psychologists. After the airplane crash, psychosocial support was officially integrated as a necessary component into the national rescue plan of Luxembourg. 

##### Establishment of Working Groups and New Units

As a central long-term impact, many different units and working groups were created after the disasters. The established working groups with experts and persons concerned analyzed past events, with the objective of developing recommendations for further improvements. The National Counter-Terrorism Coordination Centre has also been created, aiming to improve coordination between the National Police and the Guardia Civil, and terrorism experts have been sent from the Ministry of Interior to key embassies, to improve exchanges of information. After the airplane crash, an airline emergency committee was rebuilt, and a crisis-support center for Luxair accidents abroad was established. After the avalanche, the local government established a center to link research institutions, non-profit organizations, and businesses in the field of environmental hazards, to improve security measures, create databases, and develop efficient and up-to-date scenario plans.

##### Legal Changes 

Several changes in laws (fire safety regulations and railway act, which also includes funiculars) have occurred after the Kaprun tunnel fire [34]. As a response to the disaster in Galtuer, the federal minister decided to update the guidelines of the danger areas. Basics of the administration procedures are the danger-zone plans, state-specific land-use planning laws, and the forest law of 1975. As a consequence of the flood, the land registry plans have been re-evaluated, and danger zones have been updated. However, various interview partners pointed out the difficulty of keeping danger zones up in the long-term: “The danger is simply that the pressure on the administrative employees comes when nothing has happened for a long time, that one says the yellow zone is no longer necessary. Or people demand the red zone to become a yellow zone in which building, under certain conditions, is possible. The pressure is certainly there, because we are in need of soil that can be built on”. Moreover, concepts for resettlement from the endangered areas have been developed. However, it is unclear if and how many people have agreed to resettlement. A decision of the airline management after the crash in Luxembourg led to the implementation of all recommendations from aircraft designers concerning technical details in a compulsory way. 

#### 3.2.2. Infrastructure

For all investigated disasters, measures concerning infrastructure were initiated. These measures are referred to as items that improve security, in general, or specific measures to optimize processes, to be better prepared for future disasters. The costs of these infrastructure measures differ substantially.

##### Large-Scale Investments 

After the avalanche, the avalanche barriers around Galtuer were improved and extended. In order to protect the houses of Galtuer in the future, an avalanche barrier was built in the middle of the village center. This special construction, called “Alpinarium”, combines avalanche protection and an integrated museum, which deals with the history of Galtuer and the avalanche risk in the area. Further infrastructural measures were the improvement of the meteorological station, a reforestation project, and the extension of the security tunnels at the access road to Galtuer.

High investments were also undertaken in the case of the Austrian flood. Improvements and extended flood protection measures with 34 new building projects were realized. The building process had to be sped up after the flood because 17 projects had not been realized until then. Compared to the building projects, public authorities weighed the importance of the security of the public and decided not to invest in a dam, because of the complex construction project, the enormous monetary investment, and low benefit [35]. For affected public buildings like kindergartens and community buildings, the high possibility of future floods was considered, and a flood-proof way of construction and the addition of flood-resistant materials were implemented. For better forecasts and accurate prognosis, automated water-level measurement stations have been installed. However, improved technical equipment does not automatically lead to better flood protection, as one interview partner explains: “The most difficult thing is the final interpretation of the measured values. A river is a living organism, constantly changing”. The damage of the transport medium—as what took place in Kaprun—catalyzed the building of a new ropeway instead of a new funicular due to security reasons. Due to the legal adaptions, many existing funiculars in Austria had to be adapted to guarantee a high level of security for the passengers. Finally, new helicopters with a higher load capacity were purchased for the Austrian Armed Forces, to ensure access to the valleys in the event of natural hazards restricting access to the population.

##### Small- and Medium-Scale Investments

Small- and medium-sized investments were undertaken in the case of Luxembourg, to improve transport by using more containers for meeting places or restrooms for rescue teams for future operations. These decisions were based on low costs and high flexibility. Additionally, a new operation control car was acquired. In Spain, public infrastructures were controlled more intensively after the terror attack, and constructional adaptions were undertaken. Security measures, like video surveillance, emergency exits, controls of specific infrastructure, and future usage of flak vests, were implemented. 

#### 3.2.3. Research

After the Galtuer avalanche, the local government invested in research projects which should reduce future disaster risks. One project was the “Alpine Safety and Information Centre (ASI)”. The mission of this non-profit organization was to promote safety mountain environment and to act as a communication bridge between all participating institutions and local organizations. One product of these research investments that is now being used in practice is the “ESIS Tirol” mission information system, an internet platform that facilitates communication and coordination in the event of a disaster. Moreover, new calculations for avalanche simulation models have been developed. 

### 3.3. Others 

Because there were some problems in the flow of information between the response organizations, the communication processes have been updated in all investigated disasters. Moreover, technological improvements like the implementation of a uniform radio system in Tyrol and special software have been undertaken. The high media interest after the avalanche and the tunnel fire had a negative impact on the well-being and health of people affected. Therefore, improvements in media management were undertaken. Changes regarding the trainings and exercises (e.g., special topics such as media or psychosocial support, more joint trainings, and cross-border cooperation) could also be observed in all cases. Measures for improvements in the coordination of processes between the organizations and of the handling of professionals and volunteers have been initialized in all cases. Formal and informal networking was identified as a valuable basis for future cooperation. An interview partner pointed out the following: “I would add that in order to improve our response in emergencies, all the teams intervening in an emergency should have more meetings and we should learn to coordinate ourselves better, defining new plans on coordination, structure and control to make sure we all know who’s in charge, where we have to go and what we have to do”.

Table 2 and Table 3 summarize the various impacts each disaster had on the public health system.

Many of the identified impacts overlap with the recommendations in the Sendai Framework [5]: update of preparedness and contingency plans (Paragraph 33a), forecasting and early warning system (Paragraph 33b), trainings and exercises (Paragraphs 33f, 33h, 34f, and 34h), land-use planning (Paragraph 33j), provision of psychosocial support (Paragraph 33o), and revision of laws (Paragraph 33p). It can be concluded from this that the opportunity to “Build Back Better” and consequently enhance disaster preparedness has been taken.

## 4. Discussion

Based on the results of the literature review, we identified highly fragmented studies without any standardized approach to investigating the long-term impacts of disasters on public healthcare. We did not find any standard definition of long-term impact on the healthcare system. In the context of long-term impacts, the literature mainly focuses on long-term psychological impact effects on affected populations. A broader approach is missing. The studies have also chosen different time frames that do not allow any comparative conclusions.

The investigation of the case studies has shown that each disaster causes aftermaths in various fields. Learning circles [36] play a substantial role in the context of disaster management, as many of the identified long-term impacts on the public health system are the result of a learning process because of inadequate outputs in the past.

The changes observed all seem to have been sustained over the years. One exception is the ASI center, which, according to one interviewee, was closed for political reasons. The necessity of the acquired helicopters is regularly discussed in politics and media. However, landslides and roadblocks due to avalanche risk repeatedly demonstrate their importance for the protection of the affected population.

The study showed us that federalism and organizational boundaries can be a hindrance to improvements: After the 1999 avalanche in Galtuer, the Red Cross created a crisis intervention team in the province of Tyrol. In the neighboring province of Salzburg, however, there was no such infrastructure for psychosocial support yet established when the tunnel fire happened in November 2000. Christensen, Lægreid, and Rykkja (2013) describe something similar in their study: A major obstacle after the terrorist attack in Oslo was the fragmentation of responsibility within government departments. This might have hindered information-sharing and, consequently, taking measures which could have reduced the impact of the Oslo terror attack in 2011, as plans for improving the security of the building had already been established prior the attacks, but not implemented yet [37].

There is the need to analyze the individual and social circumstances of people affected. Results show the importance of analyzing long-term impacts from the perspective of the type (e.g., disaster associated with natural hazards or human-made disaster) and characteristic (e.g., duration and extent) of a disaster, as well as the regional context where a disaster took place. The effectiveness of disaster management procedures is dependent on a number of contingencies (e.g., not only how accurately one system is implemented, but also how well aligned a system is with cultural subsystems) [5,38,39]. As became apparent in Galtuer, the inhabitants applied coping strategies that are rooted in local traditions (e.g., importance of spiritual support). Nevertheless, the consequences of the avalanche led the local population to increasingly open up and cope with this specifically challenging situation by augmented communication. The ex post facto identification of local practices could be highly valuable as basis for discussion within a broader audience of special interest groups (e.g., experts for avalanche risk areas in Austria, Italy, and France).

The chosen cases include disasters caused by natural hazards, as well as human-made disasters. Both types of disasters have been demonstrated to have a potentially high impact on the public health system. However, they might have different consequences concerning preparedness planning. As avalanches and floods are often foreseeable, proactive actions like early warning and evacuation are possible in many cases. On the other hand, avalanches and floods result in relatively large impact areas, which makes response more difficult and requires thorough preparedness regarding mobilization and equipment. Differences between disasters caused by natural hazards and human-caused disasters are also observed with regards to mental health.

Dynes and Quarantelli [40] describe disasters caused by natural hazards as “consensus crises”, leading to an increase of community cohesiveness and moral, whereas human-made disasters are characterized by human blame [41]. This has consequences for the coping process and the need for psychological support. Although the disasters analyzed in the case studies may have different characteristics, they share many similarities in their impact. Recurring themes in all the case studies were infrastructural measures, update of emergency plans, changes in communication procedures, and a raised awareness for the importance of mental health and providing psychosocial support. It might prove difficult to compare different individual disaster-management cases in order to elect one best practice example. However, the use of historical lessons can be a valuable source for improvements regarding disaster preparedness [38]. Gaining insights from out-of-sector lessons is often overlooked or considered as not relevant [42]. The recurring themes we identified across the various disasters analyzed in this study, however, demonstrate the learning opportunities from other fields or kinds of disasters. Crichton et al. [42] recommend broadening the perspective and trying to apply lessons beneficially to the own environment. Therefore, the learnings from these European cases can also be of use for countries with other structures and regulations regarding their public health system and disaster management structure. Even though political, socioeconomic, cultural, environmental, and hazard circumstances vary in every state, good practices might be transferable. This learning can be achieved when using a customized approach by “making use of others’ experience, for instance by reviewing the contexts of particular measures and the nature of good practices and lessons learned, and then tailoring these to implement policies and activities that are appropriate for the local contexts” [43] (p. 5). Moreover, the Sendai Framework [5] points out the need for adaptions to the respective jurisdictions, capacities, and capabilities of each country.

Although each disaster is unique in its progress and coping, we ask for the design of a standardized assessment system for long-term disaster impacts. This would help to increase comparability of disasters. We share the recommendation expressed in the WHO Health-EDRM framework [1] regarding future research needs: a holistic all-hazard perspective across all disaster-management phases which includes physical, mental, and psychosocial needs. In order to achieve this, multidisciplinary and multi-sectoral collaboration between science, policy makers and practitioners is needed [39,44,45]. Such an exchange and dialogue between stakeholders is important in order to identify knowledge gaps, jointly develop knowledge, and, finally, to put scientific findings into practice [5].

## 5. Limitations

The chosen case-study methodology can be criticized because of its limited generalizability. The results of our case studies are a preliminary investigation with the intention of generating a first understanding of long-term impact and its underlying determinants. Although it is hardly possible to derive a holistic model covering all cumulative effects of disasters, such case studies can serve as a guide for researchers, policy makers, and disaster managers [3].

The case studies refer to disasters that have occurred in the European Union. Analyzing cases from other countries, which have different disaster management structures and health system regulations, might be an interesting task for future research offering further insights.

## 6. Conclusions

In this study, we investigated the long-term impacts of disasters on the public health system. We used a mixed-method approach consisting of document analysis and expert interviews. For our analysis, we chose the following cases: an avalanche, a cable-car accident, an airplane crash, a terror attack, and a flood. The analysis of the case studies revealed the variety of direct and indirect impacts on population health and health systems major incidents can have. We grouped the identified impacts into the categories of health-system performance, and security and health protection. Subcategories in health-system performance were mental health and demand for healthcare services, as well as structure and organization of psychosocial support. The subcategories for security and health protection were contingency and preparedness planning, infrastructure, and research. Although we chose contrasting cases, we identified recurring themes in all the cases investigated. A change in communication processes, updates of emergency plans, infrastructural measures, and a higher awareness for psychosocial support was observed in each case study. Our chosen holistic strategy gave us deep insights into each case study and helped us to better understand the undertaken or missing reactions concerning public health. By analyzing past events and their consequences on the public health system, one can develop strategies for better dealing with similar events in the future.

## Figures and Tables

**Table 1:** Overview of number of interviewees per case study.

Case Study	Number of Interviewees
Avalanche, 1999 (Austria)	7
Cable-Car Accident, 2000 (Austria)	7
Airplane Crash, 2002 (Luxembourg)	7
Terror Attack, 2004 (Spain)	7
Flood, 2013 (Austria/Germany)	8

**Table 2:** Overview of long-term impacts on health-system performance.

	Avalanche	Cable Car Accident	Airplane Crash	Terror Attack	Flood
Mental health and demand for health care services	Significance of different needs of target groups Refusal of external psychosocial support	Influence of legal proceedings on mental health and coping	Significance of different needs of target groups	Special long-term care for people with hearing impairment due to explosions needed Sign language courses for families	
Structure and organisation of psychosocial support	Establishment of KIT by Austrian Red Cross Financing structure for psychological treatment Uniforms for emergency psychologists	Establishment of KIT by Austrian Red Cross		Formation of special psychological care units	

**Table 3:** Overview of long-term impacts on security and health protection.

	Avalanche	Cable Car Accident	Airplane Crash	Terror Attack	Flood
Contingency and Preparedness Planning	Adaption of danger areas Creation of new working groups and units Update emergency plans	Update emergency plans Changes in fire safety regulations and railway act	Psychosocial support officially integrated in national rescue plan Creation of new working groups and units Update emergency plans	Creation of new working groups and units Update emergency plans Creation of new psychological protocols and plans	Re-evaluation of land registry pans
Infrastructure	Avalanche barriers Reforestation> Extension security tunnel Higher load capacity helicopters	Ropeway instead of funicular	Extended use of containers Acquisition of operation control car	More video surveillance Adaptions of emergency exits Flak vests	Flood protection building measures Flood-proof way of construction for public buildings Automated water level measurement
Research	ASI Centre ESIS mission information system Avalanche simulation models		-	-	-
